# Is the oxytocin system a keystone in human wellbeing?

**DOI:** 10.3389/fnins.2026.1989082

**Published:** 2026-10-28

**Authors:** C. Sue Carter, Hans P. Nazarloo

**Affiliations:** 1 Kinsey Institute, Indiana University, Bloomington, IN, United States; 2 Department of Psychology, University of Virginia, Charlotteville, VA, United States; 3 Department of Psychiatry, University of Florida Health Sciences, Jacksonville, FL, United States

**Keywords:** stress resilience, hormesis, oxytocin, sociostasis, wellbeing

## Abstract

It has also taken over a century for science to fully realize that oxytocin is far more than a “female reproductive hormone.” Both the measurement and interpretation of the biochemical and functional properties of oxytocin have raised controversy and remain works in progress. However, oxytocin is now recognized as a pleiotropic molecule that sits at the center of an extended system which includes vasopressin, the autonomic nervous system, the immune system and more. This larger system affects essentially every bodily function and helps to coordinate these with social context, the history of each individual and perhaps the existence of humans as a species. Together these support survival, reproduction and wellbeing, allowing mammals to manage and benefit from various forms of hormetic challenge. Here we suggest that oxytocin, viewed as a system, in turn acts as a keystone capable of connecting mammalian regulatory physiology to the benefits of social connection.

## What is oxytocin?

The molecule called “oxytocin” was identified over a century ago (Du Vigneaud et al., 1953). Oxytocin initially was implicated in birth and lactation. Oxytocin also was associated with maternal behavior (Pedersen et al., 1982; Kendrick et al., 1986). Beginning around 1970 oxytocin began to be called a “love hormone.” (Klopfer, 1971). These designations probably slowed research because they seemed to point to a sex-specific role for oxytocin. Oxytocin was later implicated in both sexes in social monogamy, parental behavior and other behaviors. However, this early research, and the abundant nomenclature that followed did not capture the functional complexity of oxytocin. Oxytocin is now understood as a critical component in an adaptive, regulatory system that extends well beyond reproduction and sociality (Hurlemann and Grinevich, 2018) (Figure 1). Among the many recognized functions of oxytocin are autonomic and cardiovascular regulation, metabolism, immunity, inflammatory processes, regulation of the microbiome, and more (Carter et al., 2020) (Figure 2).

**Figure 1:**
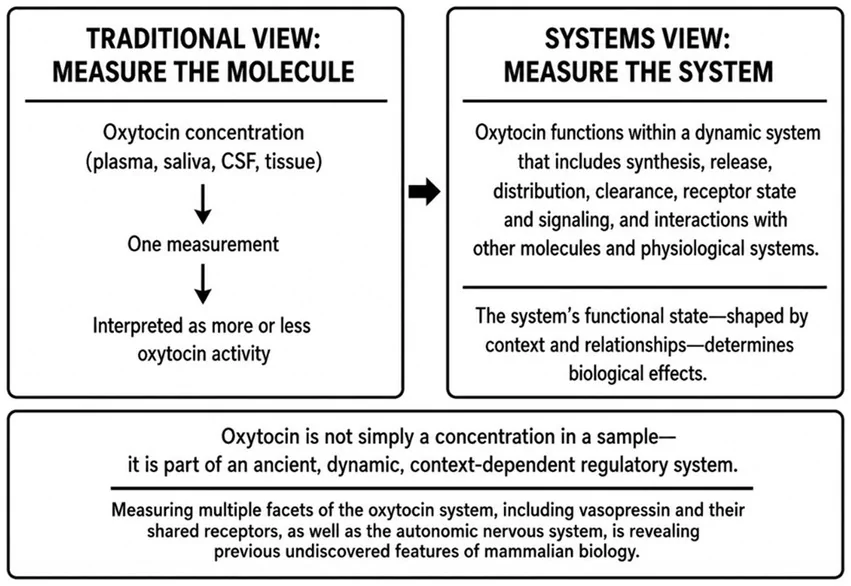
Measuring oxytocin: from a molecule to a system. The left panel illustrates a single oxytocin concentration in plasma, saliva, cerebrospinal fluid (CSF), or tissue as an indicator of biological activity. The right panel situates this measurement within a dynamic system of synthesis, release, distribution, clearance, receptor state, and signaling. The lower panel highlights complementary measures of vasopressin, receptor interactions, and autonomic function.

**Figure 2:**
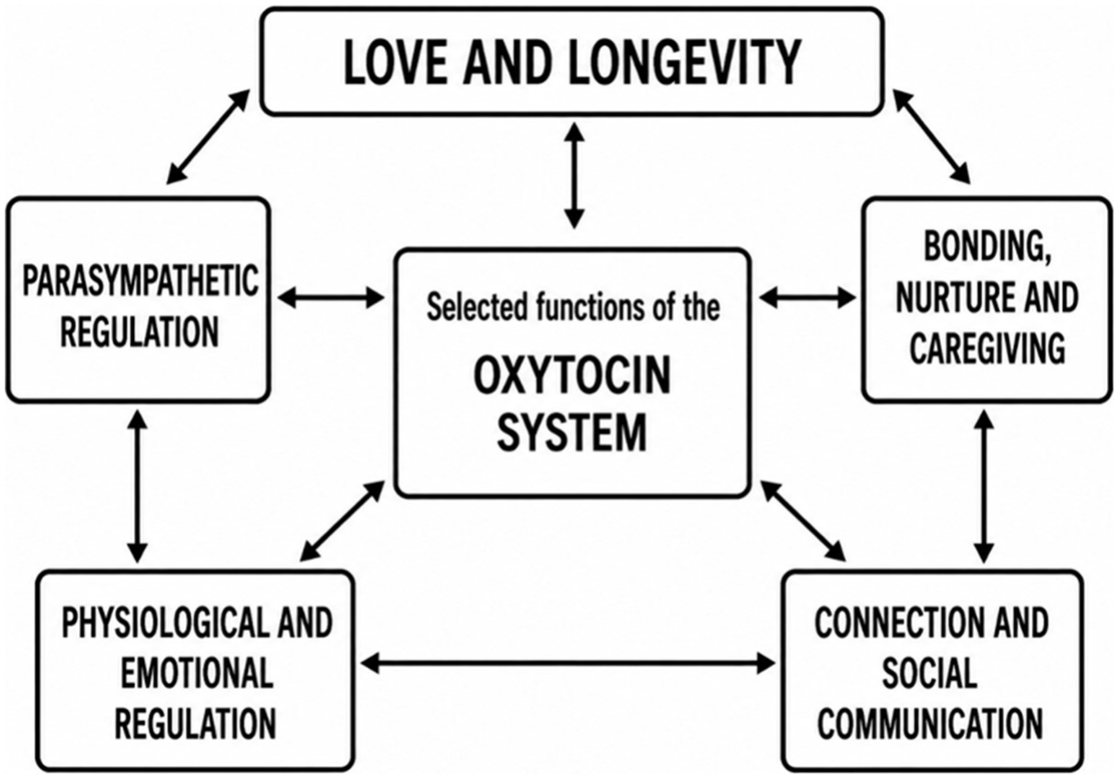
Selected functions of the oxytocin system and their proposed relationships with love and longevity. Bidirectional arrows indicate reciprocal relationships between these domains and the oxytocin system, linking social behavior with physiological regulation. These interactions illustrate sociostasis, the regulation of physiological state through social interaction, and its potential contributions to wellbeing and longevity.

Oxytocin in its classical form emerged coincident with the evolution of mammals and nurture directed toward offspring (Grinevich et al., 2016; Kendrick et al., 1986). The effects of oxytocin are most apparent in the face of a challenge, sometimes gaining for oxytocin the title of an “anti-stress hormone” (Neumann, 2002; Nazarloo et al., 2025). Oxytocin also plays a major role in the management of inflammation (Uvnas Moberg et al., 2019) and oxidative stress, appearing as a component of what we have previously suggested might be a “social solution to the stress of life”. These fundamental properties of the oxytocin system may help to explain the repeatedly described benefits of social factors in wellness and eventually longevity (Horn and Carter, 2021).

## A keystone in a “bridge over troubled waters”

Oxytocin and its receptors have the capacity to coordinate multiple processes across brain and body. In fact this remarkable position in mammalian biology extends the influence of oxytocin beyond the individual, supporting the general benefits of sociality to a regulatory process we have termed “sociostasis”. Oxytocin also may begin to explain previously unrecognized mechanisms underlying various benefits that follow manageable stress - a phenomenon that has been termed “stress-response hormesis” (Calabrese and Mattson, 2024). In this context, we suggest that the oxytocin system might be considered a regulatory “keystone” in mammalian sociality, functioning to facilitate survival, wellbeing, love and longevity.

A keystone is not the entire structure and does not act independently of the structure around it. Its significance comes from its position and its relationships with other components. Oxytocin sits at the center of an ancient system, capable of supporting mammalian physiology and longevity through effects on both physiology and behavior. Challenge and adversity can contribute to either dysregulation and disease or adaptation and resilience. The oxytocin system, through its reciprocal relationships with social connection and physiological regulation, may help shape this balance. Sociostasis can provide a regulatory context in which manageable challenge becomes hormetic, supporting wellbeing, resilience, and ultimately love and longevity. These and other functions of oxytocin may help to explain what is sometimes called “the healing power of love.”

## Context and the future

The challenge forward is both analytical and conceptual. Of course, accurate measurements remain essential. But as methods improve, their greatest value may come from recognizing that they are measuring different facets of a dynamic regulatory system (Nazarloo, this volume). Rather than seeking one definitive measure of oxytocin, we may need to find multiple valid measures to reveal how the oxytocin system is organized, regulated, and functionally engaged.

This approach turns a longstanding measurement problem into an opportunity. The apparent complexity of oxytocin measurement, which has been a repeated source of controversy, may not simply be methodological noise. Properly studied, it may help reveal previously undiscovered properties of one of mammalian biology’s most ancient and broadly integrated regulatory systems.

This discussion is not intended to diminish the value of attempts to measure or describe specific features of the oxytocin molecule (Lawson, 2026). An extensive literature, based in large part on peptide concentrations, has established much of what is known about oxytocin biology. Despite the admitted limitations of current measurements, double-blind studies, in both human and nonhuman models, have repeatedly discovered statistically and biologically-significant relationships between endogenous and exogenous oxytocin and various diverse functions. Awareness of oxytocin’s functions also has offered insights into human evolution. Moreover, understanding the broader functions of the oxytocin system as a central element in mammalian biology reframes our understanding of the deep biology of factors and processes that can contribute to human health and wellbeing.

## Data Availability

The original contributions presented in the study are included in the article/supplementary material, further inquiries can be directed to the corresponding author.
